# Surgical treatment of acute fingernail injuries

**DOI:** 10.1007/s10195-011-0161-z

**Published:** 2011-10-08

**Authors:** P. Tos, P. Titolo, N. L. Chirila, F. Catalano, S. Artiaco

**Affiliations:** 1 Department of Orthopedics and Traumatology, UOD Reconstructive Microsurgery, CTO-M. Adelaide, Via Zuretti 29, 10126 Turin, Italy; 2 Department of Surgical Specialities, UOD Plastic and Reconstructive Surgery, University of Messina, Messina, Italy

**Keywords:** Fingernail injury, Nail surgery, Fingernail repair

## Abstract

The fingernail has an important role in hand function, facilitating the pinch and increasing the sensitivity of the fingertip. Therefore, immediate and proper strategy in treating fingernail injuries is essential to avoid aesthetic and functional impairment. Nail-bed and fingertip injuries are considered in this review, including subungual hematoma, wounds, simple lacerations of the nail bed and/or matrix, stellate lacerations, avulsion of the nail bed, ungual matrix defect, nail-bed injuries associated with fractures of the distal phalanx, and associated fingertip injuries. All these injuries require careful initial evaluation and adequate treatment, which is often performed under magnification. Delayed and secondary procedures of fingernail sequelae are possible, but final results are often unpredictable.

## Introduction

**Fig. 1:**
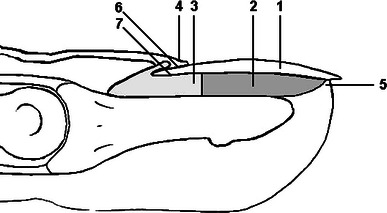
Anatomy: *1* nail plate, *2* nail bed, *3* nail matrix, *4* eponychium, *5* hyponychium, *6* proximal nail fold, *7* nail root

Fingernails have an important role in hand function. They protect the dorsal surface of the distal phalanges of the fingers and increase sensitivity of the fingertip. The fingernails facilitate the pinch of small objects, allow scratching, and have a fundamental cosmetic role. In order to plan appropriate treatment of traumatic nail injuries, careful knowledge of nail anatomy and physiology is required. The fingernail consists of the nail plate (a horny texture structure 0.5-mm thick) and the surrounding structures or perionychium. The paronychia describes the soft tissues of the lateral parts of the nail; the eponychium is the superficial dorsal roof of the superficial nail fold; the hyponychium describes the area between the nail bed and the fingertip under the free edge of the nail. The upper part of the nail fold is called dorsal roof and the lower the ventral floor. The nail bed is the part to which the nail adheres. It can be divided in two parts: the proximal part is the germinal matrix and the distal one the sterile matrix. The junction between these two parts is situated at the level of the lunula. Production of ungual keratin is attributed only to the germinal matrix, whereas the sterile matrix provides only the adherence function. The anatomy of the fingernail is described in the Fig. 1. The network of blood and lymphatic vessels is highly extended in the nail bed, and the presence of a large number of anastomoses allows the use of nail-bed and/or matrix flaps with good results during surgery. Nail generation depends on the patient’s age, gender, and habits, and the nail growth rate is approximately 0.1 mm/day (0.5 mm/week) [1].

From the epidemiological point of view, most fingernail injuries are caused by crush trauma and involve children and young adults [2–4]. In about 50% of cases, fingernail injuries are associated with phalangeal fractures. When a trauma occurs, nail generation ceases for about 21 days. Following this phase, an increase in growth rate is observed for the next 50 days and a decrease is noted for 30 subsequent days. Nail growth is normal after 100 days following trauma [1]. During this period, a transversal thickening of the nail represents signs of the pre-existing trauma (line of Beau).

In primary care, it is of great importance to achieve a smooth nail bed without scars. The wound therefore must be accurately sutured in order to prevent secondary deformities. A scar on the dorsal roof leaves an opaque streak on the nail plate; a germinal matrix scar leaves a split or no nail growth; if the scar is on the sterile matrix, a split or detachment of the nail may occur distal to the injury. The final result should be evaluated 1 year after the trauma.

## Principles in general treatment of acute nail-bed injuries and nail avulsion

Optical means magnification and a 6-0 or 7-0 nonchromic absorbable monofilament are necessary for nail-bed sutures. The nail is raised by using scissors or a delicate spatula starting under the free edge of the nail. It is carefully detached from the nail bed and, if necessary, the nail is removed from the nail fold by rotational movements [5]. The nail will be preserved and replaced like a biological dressing. This has different functions: to shape to the nail-bed fragments, to avoid adhesion between the roof and the nail bed, to support a possible associated fracture, like a splint, to decrease postoperative pain, and to improve tactile sensation during the healing period. Before replacing the nail, a few holes should be made to allow blood drainage. The nail should be firmly fixed at the end of the operation. Good insertion of the nail base into the sack bottom of the proximal nail fold is very important to prevent a dead space that may cause adherence between the nail matrix and eponychium and subsequent ungual dystrophy. To hold the nail into the nail fold, an X suture is preferable, avoiding passage through the nail bed with a U suture [6, 7]. While the new fingernail is growing, the nail used as a splint will be pushed off and substituted in 1–3 months. It is desirable not to remove the nail or the substitute so as to prevent the nail bed drying our. Steri-Strips may help keep the nail in situ during fingernail regrowth.

## Subungual hematoma

Treating subungual hematoma depends on the type of injury. When the hematoma is very small and not too painful, it is incorporated into the nail and progressively migrates to the free edge of the nail plate. Greater hematomas, involving up to 50% of the nail bed, should be evacuated through two holes made in the nail plate (in asepsis, not necessarily with anesthesia) with a needle, a blade, or an incandescent clip [8]. The pressure of the hematoma under the nail causes evacuation of the blood, allowing reinsertion of the nail into the nail fold. Steri-Strips may eventually fix the nail in order to avoid dislocation. When >50% involvement of the nail plate is associated with a fracture of the distal phalanx, examination of the nail bed is suggested. The fingernail should be detached, the hematoma drained, and the nail lesions should be identified and eventually treated [4, 9].

## Fingernail avulsion and nail substitute

**Fig. 2:**
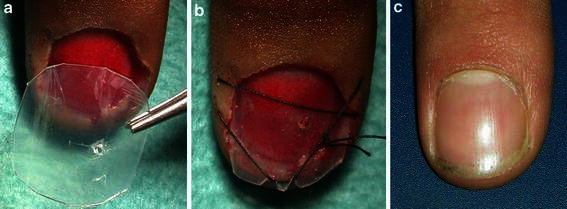
**a** Fingernail avulsion, **b** polypropylene substitute, **c** clinical result at 12 months

As mentioned, if the avulsed nail is present, it must be replaced in the nail fold. Sometimes the fingernail may be too damaged to be repositioned. In these cases, a nail substitute should be used to protect the fingernail during the healing process and to avoid adherences along the proximal nail bed and nail fold. Soft devices such as nonadherent gauze or a polyurethane sponge have been used [10, 11], but they may not protect the nail bed from pressure and pain. Others simple fingernail substitutes, such as a piece of X-ray film or a piece of the suture envelope, have been used [12, 13] but they risk being nonsterile or not effective in nail-bed protection. Prosthetic splints (INRO Surgical Nail) are described by Ogunro [14], but they are expensive and often not immediately available during surgery. For these reasons, we use a flexible polypropylene foil simply obtained by trimming the reservoir of a common infusion set [15]. This substitute is sterile, inexpensive, and easily available in emergency and elective operatory theater. The foil is strong enough to protect the nail bed during the healing period and until new fingernail growth. Furthermore, the fingernail substitute is flexible and can be shaped and adapted to the nail-curvature radius. In our clinical experience, we obtained optimal clinical results without evidence of complications (Fig. 2a–c).

## Wounds and lacerations of the nail bed and/or matrix

**Fig. 3:**
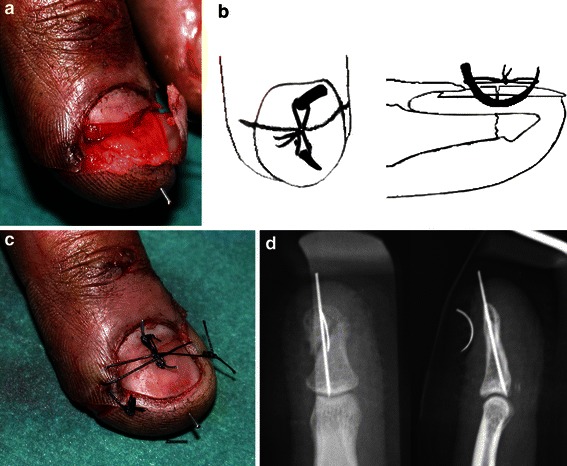
**a** Transversal nail-bed lesion, **b** nail haubanage, **c** postoperative nail haubanage, **d** postoperative nail haubanage X-ray

In case of transversal wounds with discontinuity of the nail and nail bed, synthesis can be made with a 3/8 needle passing through the nail plate and bed. A nylon suture passed around the needle may compress the nail plate against the nail bed, bringing the two parts of the nail bed into the correct position (Fig. 3a–d).

In all other cases, the nail bed may be raised and evaluated under magnification in order to carefully examine the characteristic of the lesion. If only the sterile matrix is damaged, the nail should be left attached proximally in the nail fold. When the nail germinal matrix is also involved, the entire nail should be detached by making two incisions on the lateral side of the nail fold. The nail should be left attached distally when dislocation of the proximal part of the nail occurs.

**Fig. 4:**
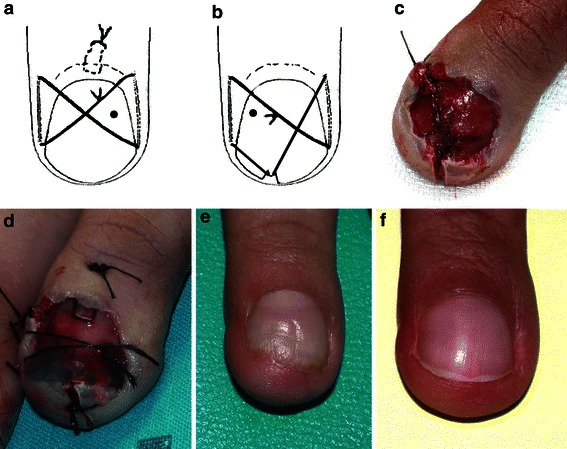
**a** X-shaped suture, **b** U-shaped suture, **c** nail-bed suture, **d** nail-substitute X-shaped suture, **e** clinical result at 4 months, **f** clinical result at 12 months

Nail-bed examination should be performed under local anesthesia, and every nail-bed injury should be repaired after nail removal. When the nail bed is intact, the nail should be reinserted into the nail fold and the lesion treated as an extensive hematoma with holes for drainage [4, 16]. Any irregularities of the wound edges of the nail bed or matrix should be avoided. The nail bed or matrix should then be approximated with 7-0 absorbable suture, with the knots placed at a sufficient distance to avoid excessive tension. A hole in the nail plate should always be made before replacement in order to allow serum and blood drainage. The nail is finally inserted in the nail fold and kept adherent to the nail bed by a 3-0 external figure U or X suture (Fig. 4a–d). When the fingernail is lost, a nail substitute is applied.

The dressing is changed every 5–7 days and the nail checked for subungual seroma or hematoma. If present, the hole is reopened to allow drainage. The suture is removed after 2–3 weeks. The nail adheres to the nail bed within 1–3 months until pushed off by the new nail, which will reach complete growth at 4–6 months after trauma (Fig. 4e, f). Treatment for simple and stellate lacerations of the fingernail is similar. In case of nail-bed or matrix injury, all fragments are preserved and replaced as free grafts in order to attain an optimal final result.

## Nail-bed avulsion (sterile and germinal matrix defect)

As a general principle, when the nail bed is avulsed, it should be always repositioned to obtain anatomical reconstruction of the fingernail. Thus, when a fragment of the nail bed remains attached to the undersurface of the avulsed nail, it should be replaced as a composite free graft. If the avulsed fragment is not available because of loss or destruction, conservative treatment or reconstructive techniques can be considered. Conservative techniques are based on the observation that the nail bed has a regenerative potential that allows for complete nail repair in about 6 weeks [17]. In his study, Ogunro [14] reported that when the residual nail bed is effectively covered, in order to prevent drying and maintain a local environment suited for tissue regeneration, normal nail growth may be obtained.

Reconstructive techniques can be used when larger nail-bed defects are observed, but these procedures may be demanding and not immediately executable in all the orthopedic and plastic surgery centers. There are several options for reconstructing sterile and matrix defects, including split-thickness or full-thickness grafts, rotational flaps, and composite grafts. The choice of donor site is made according to the extent of the lesion. It is possible to select: (a) nail bed from uninjured areas of the involved finger; (b) a bank finger when the injured finger is not available for replantation; (c) uninjured fingers or the big toe for larger defects (it may be harvested in an emergency even under local anesthesia). Split-thickness nail-bed graft may be harvested from uninjured areas of the involved finger if the defect is small or from adjacent uninjured finger or toe when larger nail-bed areas are involved. Nail-bed graft can be placed directly on the exposed cortex of the distal phalanx, sutured to the surrounding nail bed, and appropriately dressed [18]. Full-thickness nail-bed grafts have the disadvantage of causing deformity of the donor site and are rarely used except when there are salvageable spare parts that would otherwise be not used [19]. A full-thickness nail-bed graft is necessary, however, when replacing lost germinal matrix to support regeneration of the nail plate or in case of complex injury of the perionychium surrounding the nail bed [20].

The well-vascularized nail bed and matrix enable the use of rotation flap as a proximal or distal pedicled flap for large defects (even 5–6 mm) or bipedicled flap for defects <3 mm. For more complex injuries, some authors suggest nonvascularized composite tissue grafts, combining sterile and germinal matrix and eponychium, usually performed from the second toe. Only 50% have been described as attaining good results, and donor-site sequels are not negligible. Many techniques and variations from the wrap-around flap of Morrison have been reported to allow reconstruction in one setting of combined bone and soft-tissue loss. In those cases, the pedicles are sutured at the level of the proximal interphalangeal joint.

## Fractures of the distal phalanx and fingertip injuries associated with nail-bed wounds

Approximately 50% of nail-bed injuries have an associated fracture of the distal phalanx [4]. In nondisplaced fractures, nail-bed repair and nail replacement (which acts as a splint) with a tension-band suture may allow optimal stability [21]. As an alternative technique, Kirschner-wire fixation with a tension band suture may be used [7]. Unstable displaced fractures should be reduced and fixed with fine longitudinal or crossed Kirschner wires. In distal fractures, a 21-gauge needle can substitute for the 0.8-mm Kirschner wires. In phalangeal fractures associated with transversal injuries of the nail plate, the haubanage should be performed with a needle, as described above. The hyponychium reconstruction is important to avoid hooking the nail. Many local flaps have been described for fingertip loss of substance, such as V–Y advancement flap, Hueston flap, and Venkataswami flap [22–24]. The distal part of the flap should be fixed to the bone with an intradermic needle to avoid hook deformity [25, 26]. This fixation discharges the forces of the flap directly to the bone, preserving the nail bed and avoiding a secondary hooking nail deformity. In these cases, no stitches are necessary between the flap and the nail bed.

## Conclusion

Nail injuries result from crushing trauma that causes compression of the nail to the subjacent bony surface. The pattern of fingernail injury depends on the energy and direction of trauma. Various types of injuries can be described, including subungual hematoma, simple injuries of the nail bed and matrix, lacerations and contusions, more complex injuries associated with tissue loss with or without avulsion, and/or associated fractures. Management of a fingernail injury should be selected on the basis of injury type and extent, and requires accurate knowledge of nail anatomy and physiology. An effective emergency treatment is mandatory to prevent secondary deformities and reduce the risk of secondary reconstruction of the nail bed, which often gives unpredictable results.
